# Efficacy of ULV and thermal aerosols of deltamethrin for control of *Aedes albopictus* in nice, France

**DOI:** 10.1186/s13071-016-1881-y

**Published:** 2016-11-23

**Authors:** Saïd C Boubidi, David Roiz, Marie Rossignol, Fabrice Chandre, Romain Benoit, Marc Raselli, Charles Tizon, Bernard Cadiou, Reda Tounsi, Christophe Lagneau, Didier Fontenille, Paul Reiter

**Affiliations:** 1 EID Méditerranée, Montpellier, France; 2 Service Eco-épidémiologie parasitaire, Institut Pasteur, Algiers, Algeria; 3 UMR Mivegec, IRD, CNRS et Université de Montpellier, Montpellier, France; 4 Institut Pasteur du Cambodge, Phnom Penh, Cambodia; 5 Unité Insectes et Maladies Infectieuses, Institut Pasteur, Paris, France

**Keywords:** *Aedes albopictus*, ULV, Cold fogging, Thermal fogging, Vector control, Dengue, Deltamethrin

## Abstract

**Background:**

Ultra-low volume (ULV) insecticidal aerosols dispensed from vehicle-mounted cold-foggers are widely considered the method of choice for control of *Aedes aegypti* and *Ae. albopictus* during outbreaks of dengue and chikungunya and, more recently, Zika. Nevertheless, their effectiveness has been poorly studied, particularly in Europe. Nearly all published studies of ULV efficacy are bio-assays based on the mortality of caged mosquitoes. In our study we preferred to monitor the direct impact of treatments on the wild mosquito populations. This study was undertaken to evaluate the efficiency of the two widely used space spraying methods to control *Ae. albopictus* and *Ae. aegypti*.

**Methods:**

We determined the susceptibility of local *Ae. albopictus* to deltamethrin by two methods: topical application and the “WHO Tube Test”. We used ovitraps baited with hay infusion and adult traps (B-G Sentinel) baited with a patented attractant to monitor the mosquitoes in four residential areas in Nice, southern France. The impact of deltamethrin applied from vehicle-mounted ULV fogging-machines was assessed by comparing trap results in treated *vs* untreated areas for 5 days before and 5 days after treatment. Four trials were conducted at the maximum permitted application rate (1 g.ha^-1^). We also made two small-scale tests of the impact of the same insecticide dispensed from a hand-held thermal fogger.

**Results:**

Susceptibility to the insecticide was high but there was no discernable change in the oviposition rate or the catch of adult female mosquitoes, nor was there any change in the parous rate. In contrast, hand-held thermal foggers were highly effective, with more than 90% reduction of both laid eggs and females.

**Conclusions:**

We believe that direct monitoring of the wild mosquito populations gives a realistic assessment of the impact of treatments and suggest that the lack of efficacy is due to lack of interaction between the target mosquitoes and the ULV aerosol. We discuss the factors that influence the effectiveness of both methods of spraying in the context of epidemic situations.

**Electronic supplementary material:**

The online version of this article (doi:10.1186/s13071-016-1881-y) contains supplementary material, which is available to authorized users.

## Background

Dengue is transmitted by two species of mosquito, *Aedes aegypti* and *Ae. albopictus*, that thrive in the urban environment. For this reason it is a predominantly urban disease. In the past 50 years, the incidence and prevalence of dengue have risen dramatically; it is now by far the most important arboviral disease and a major public health problem throughout most tropical and some sub-tropical regions worldwide. According to the most recent estimates, *c*.390 million people are infected each year and 96 million manifest with clinically apparent illness [1]. Two other arboviral diseases, chikungunya and Zika, are transmitted by the same vectors and have achieved high profile in the past decade after major urban outbreaks in the Americas, Africa and Polynesia [2].

The tiger mosquito *Ae. albopictus*, native to Asia, was first detected in Europe in the 1970s [3] and is now widespread, often common in at least 18 countries. An outbreak of chikungunya (292 clinical cases) in northeastern Italy [4] confirmed that epidemic transmission of this virus is possible. Sporadic autochthonous cases of dengue and chikungunya associated with infected travellers continue to occur at various sites in Mediterranean Europe, particularly in France [5–7], a forewarning of future outbreaks [8]. It is likely that the northward progression of the vector will expand the geographic range of such events. Moreover, Zika virus epidemics occurring currently in the Americas raise concerns, though this virus is adapted not only to *Ae. aegypti* and *Ae. albopictus* but to several species of mosquito [9, 10].

In the absence of a vaccine, vector control is the only option for suppression of transmission. In many countries, ultra-low volume (ULV) insecticidal aerosols dispensed from vehicle-mounted cold-foggers, widely used to combat nuisance species, are considered the method of choice during public health emergencies [11]. Nevertheless, field trials have failed to demonstrate any significant impact on urban *Ae. aegypti* populations and there is no evidence that such treatments have any marked impact on epidemic transmission [12]. Moreover, even if ULV were to achieve a major reduction of adult mosquitoes, the effect would probably be too transient for any marked reduction of transmission [13].

*Aedes aegypti* is an endophilic species that spends much of its time sequestered in sheltered sites indoors, typically among clothes in closets. The disappointing impact of ULV treatments on *Ae. aegypti* may be attributable to this behavior: once launched from the machine, aerosol particles are at the mercy of air movements to deliver them to the target, yet that target is cloistered in sites that are devoid of air movement. By contrast, because *Ae. albopictus* is markedly exophilic, we were optimistic that this species would be more vulnerable to outdoor treatments. In this article we report on a series of six field trials in which we used ovitraps and B-G Sentinel traps to monitor the impact of ULV deltamethrin on wild populations of *Ae. albopictus* in residential areas in Nice, France. We also present results of small-scale treatments by hand-held thermal fogger.

## Methods

### Mosquitoes

Adult *Ae. albopictus* were obtained by rearing eggs collected in ovitraps baited with seven day-old hay infusion [14]. Larvae (200 per liter) were fed ‘Tetramin’ fish food (Tetramin Tropical Flakes-Spectrum Brands, Inc). F_0_ females were fed on cattle blood through a pig intestine membrane with the Hemotek membrane feeding system (Hemotek®). A 10% honey solution was available at all times except for 24 h before the blood meal. Three-five day-old F_1_ females were used in all assays.

### Insecticide susceptibility

Susceptibility of F_1_ *Ae. albopictus* females was determined by the standard WHO Bioassay [15] and by topical application. For the WHO test, technical grade (TG) deltamethrin 99.8% (Sigma-Aldrich, France) was diluted in acetone with silicone oil as the carrier. Eight concentrations of deltamethrin ranging from 0.0005 to 0.05%, active ingredient were used. For each replicate, four batches of 25 non-blood-fed females (2–4 day-old) were held in the exposure tubes for 30 min. Knockdown (Kd) was recorded every 5 min. Recovery tubes were maintained at 27 ± 2 °C and 80 ± 10% relative humidity with a small pad saturated with 10% honey solution. Mortality was recorded 24 h after exposure. For each concentration, a batch of 25 mosquitoes of a susceptible strain originating in French Polynesia (Bora Bora) was used as a control.

For tests by topical application, eight doses of deltamethrin (0.0013753 ng/mg to 0.0880281 ng/mg) diluted in acetone were used to provide a range of mortality from 0 to 100%. Two-five day-old non blood-fed females were anaesthetized with carbon dioxide for 60 s and transferred to a refrigerating plate at 4 °C. Insecticide solution (0.1 μl at the required concentration) was deposited on the upper pronotum by microcapillary. Mosquitoes were then transferred to plastic cups and maintained at (27 ± 2 °C) and humidity (80 ± 10%). Mortality was recorded 60 min after dosing and again after 24 h.

### Trial sites

The ULV trials were conducted in gated communities in Nice, southern France, each with a close network of roads linking approximately 200 houses. Vegetation, largely a wide range of evergreen shrubs and trees, was abundant and meticulously maintained. In the dry Mediterranean summer, mosquito breeding sites were very hard to find, yet *Ae. albopictus* was plentiful, attacking in large numbers in many shaded sites. The two most frequent breeding sites of *Ae. albopictus* were man-made containers, particularlyflower pot saucers and catch basins.

**Fig. 1:**
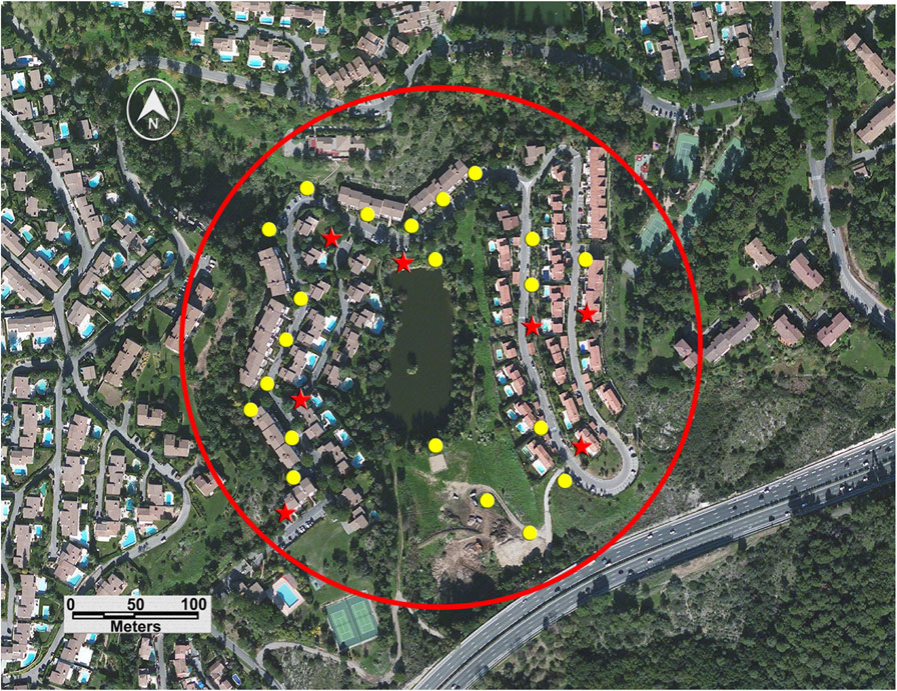
Control site: Vaugrenier Presidence, Villeneuve Loubet, Alpes Maritimes. *Stars* indicate BGs traps; *dots* indicate ovitraps

**Fig. 2:**
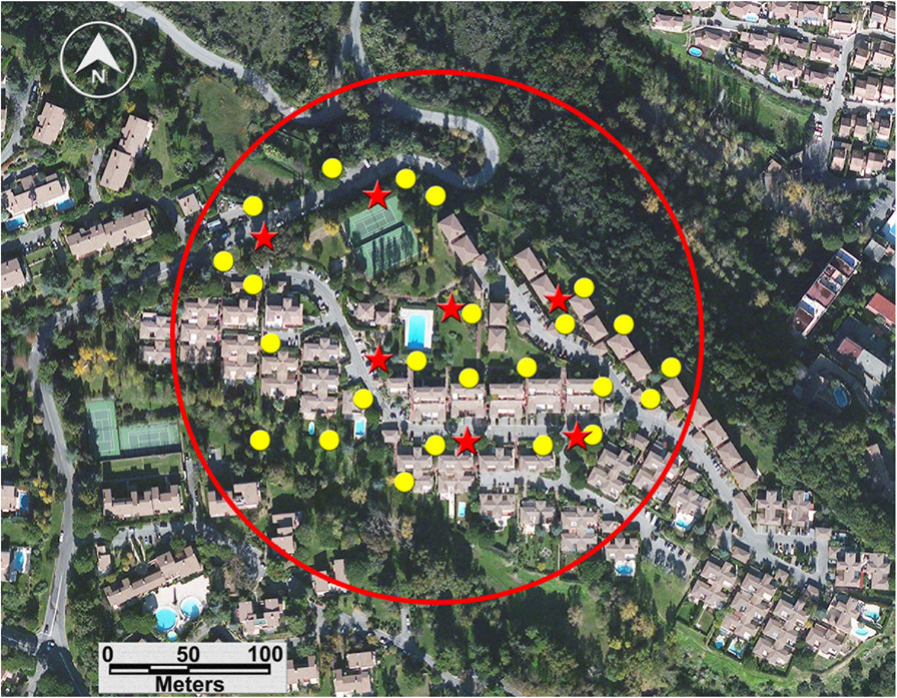
ULV treatment evaluation site, Test n°1: les Ambassades residence. *Dots* indicate ovitraps; *stars* indicate BG sentinel traps

**Fig. 3:**
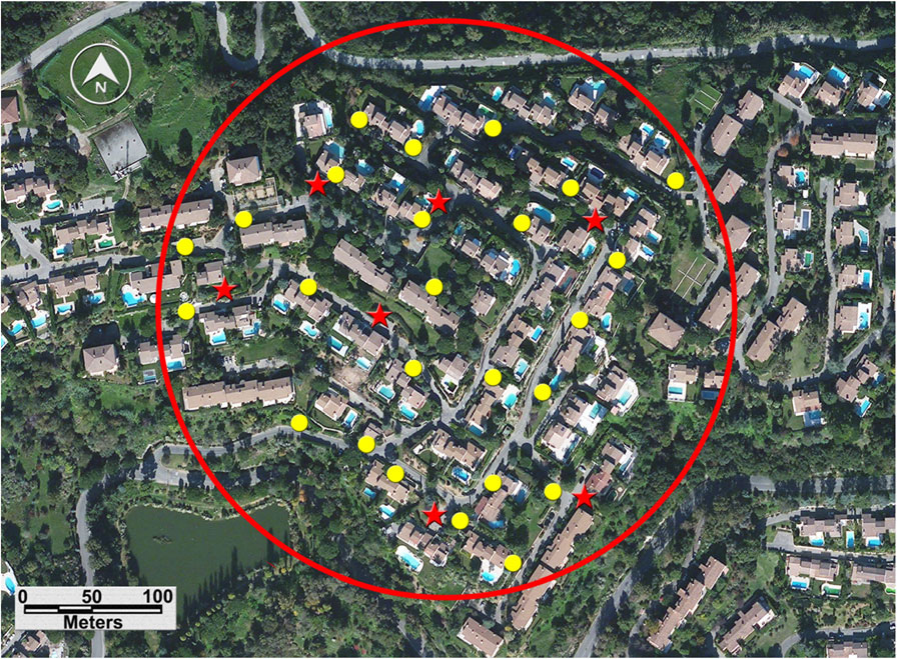
ULV treatment evaluation site, Test n°2: “La Soubrane” residence. *Dots* indicate ovitraps; *stars* indicate BG sentinel traps

The first three were in a residence in the commune of Villeneuve-Loubet in the department (= county) of Alpes-Maritimes, southeast France (Fig. 1). The fourth was in a residence with similar layout, about 350 m from the first one (Fig. 2). A third residence in the same residential area, about 500 m from the two other sites, was used as an untreated control (Fig. 3).

**Fig. 4:**
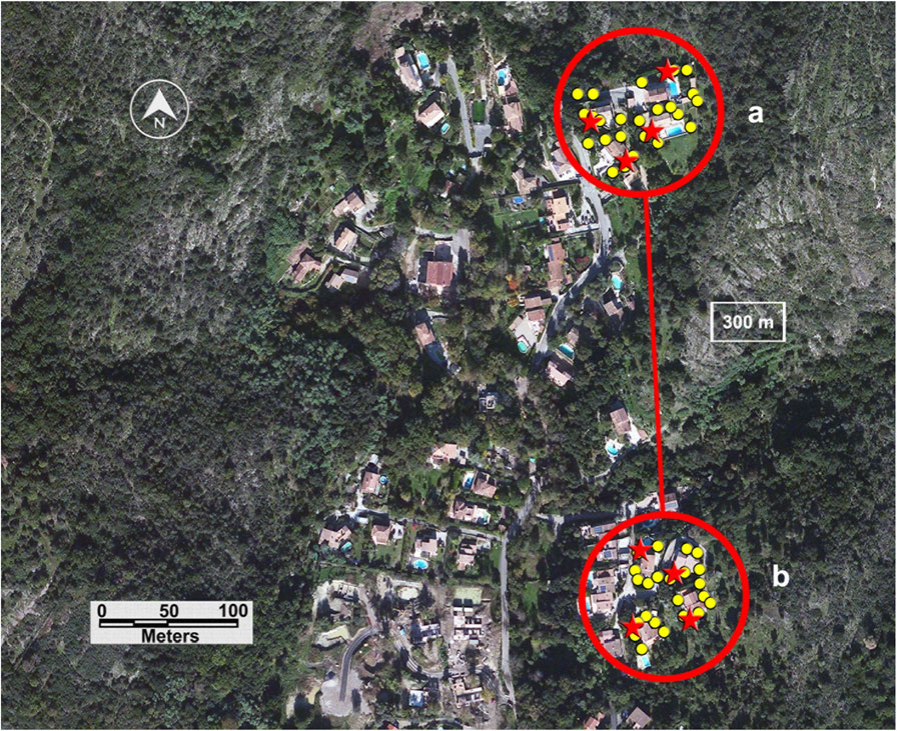
Thermal fogging evaluation site, Les Terrasses de Saint Julien. **a** Treated area. **b** Control area. *Dots* indicate ovitraps; *stars* indicate BG sentinel traps

Spraying using hand-held thermal fogger was applied in an isolated cluster of four private houses surrounded by woodland and located in the same department at Saint-Julien district. The control site includes the same number of grouped and isolated houses located at approximately 300 m from the treated site (Fig. 4).

### Insecticide formulation

Aqua K-Othrine^®^ (Bayer SAS), containing 2% deltamethrin was used at maximum permitted dose (1 g active ingredient per hectare). The product is a patented formulation (*Film Forming Aqueous Spray Technology*) that forms a protective film around the spray droplets, thereby suppressing evaporation. Our trials were performed with Aqua K-Othrine^®^, aqueous emulsionnable FFAST formulation, this new formulation (at a dose of 1 g AI/ha) were reviewed by WHOPES which mentioned a mortality of 86% on *Ae. albopictus* in caged bioassays up to 50 m downwind from the point of spray discharge, which demonstrated the good adulticidal efficacy of Aqua K-Othrine [16].

### Pre-trial preparation

Local regulations require that all residents receive a letter outlining the objectives of the trial, the date and time of the treatment, as well as safety measures (e.g. closed windows) that should be respected. On the eve of the treatment, further information was distributed in flyers and stickers, and by the management of the residence.

### Fogging equipment

Cold fogging was by a vehicle-mounted London Fogger Model 18-20 (London Foggers, Long Lake, MN, USA) ULV aerosol generator with nozzle horizontal, parallel to the road. Liquid flow-rate was 0.5 l/min, 80% of droplets below 20 μm and vehicle speed was held below 12 km/h. The solution was applied at 2 l per hectare, equivalent to 1 g active ingredient with the maximum permitted dosage. Additional file 1: Figure S1, Additional file 2: Figure S2 and Additional file 3: Figure S3 show the route of spraying in the two different residential areas. All treatments were made punctually in late dawn, one hour after astronomic sunrise.

Thermal fog was applied by a portable Pulsfog^®^ K-10-SP (GRID) (Pulsfog Dr. Stahl & Sohn GmbH) with a liquid flow rate of 0.5 l/min and 100% of droplets below 25 μm diameter. Protective clothing and safety procedures followed WHO recommendations [17]. Treatments were made all around each house, with fog mainly directed less than 1 m above the ground, with particular attention to vegetation. Treatment dates and times are summarized in Table 1.

**Table 1:** Trial dates and treatment times

Trial	Treatment	Date	Treatment time	Comment
1	ULV	26/08/2013	1 h pre-sunrise^a^	Site 1
2	ULV	24/09/2013	2 h post-sunrise^b^	Site 1
3	ULV	23/06/2014	2 h post-sunrise	Site 1
4	ULV	23/07/2014	2 h post-sunrise	Site 2^c^
		28/07/2014	2 h post-sunrise	
5	Thermal fog	09/10/2013	2 h post-sunrise	Site 3
6	Thermal fog	20/08/2014	2 h post-sunrise	Site 3

^a^Timing to minimize exposure of residents

^b^Timing to coincide with maximum flight activity period of targeted mosquitoes

^c^Site with denser network of roads enabling improved accessibility to machine

### Monitoring the mosquito population

Mosquito populations were monitored with 25 ovitraps baited with hay infusion and seven adult traps baited with a patented attractant (BG-Sentinel^®^ traps, BioGents, Regensburg, Germany). Traps were exchanged every 24-h. The thermal fog tests were on a much smaller scale, with 1 BG-Sentinel^®^ trap and 6 ovitraps in each of the four private houses in the treatment and the control clusters.

### Statistical analysis

The dose-mortality response was assessed by the R-script BioRssay [18]. This computes the doses of insecticides killing 50% and 95% of the tested colony or strain. After developing a protocol for data exploration [19], a Generalized Linear Mixed Model (GLMM) was applied with negative binomial distribution or zero-inflated negative binomial (as the data were over-dispersed) using the *glmm* ADMB package [20]. The response variables were *Ae. albopictus* female and egg abundance and parity rates. Explanatory variables are Control/Treatment and Pre/Post treatment and the interaction of both, while random variable was trap. Significant values were corrected by False Discovery Rate. Statistical analysis was performed in R version 2.14.2 [18]. The interpretation of the variables used in our statistical analysis is summarized in Additional file 5: Table S1.

## Results

**Fig. 5:**
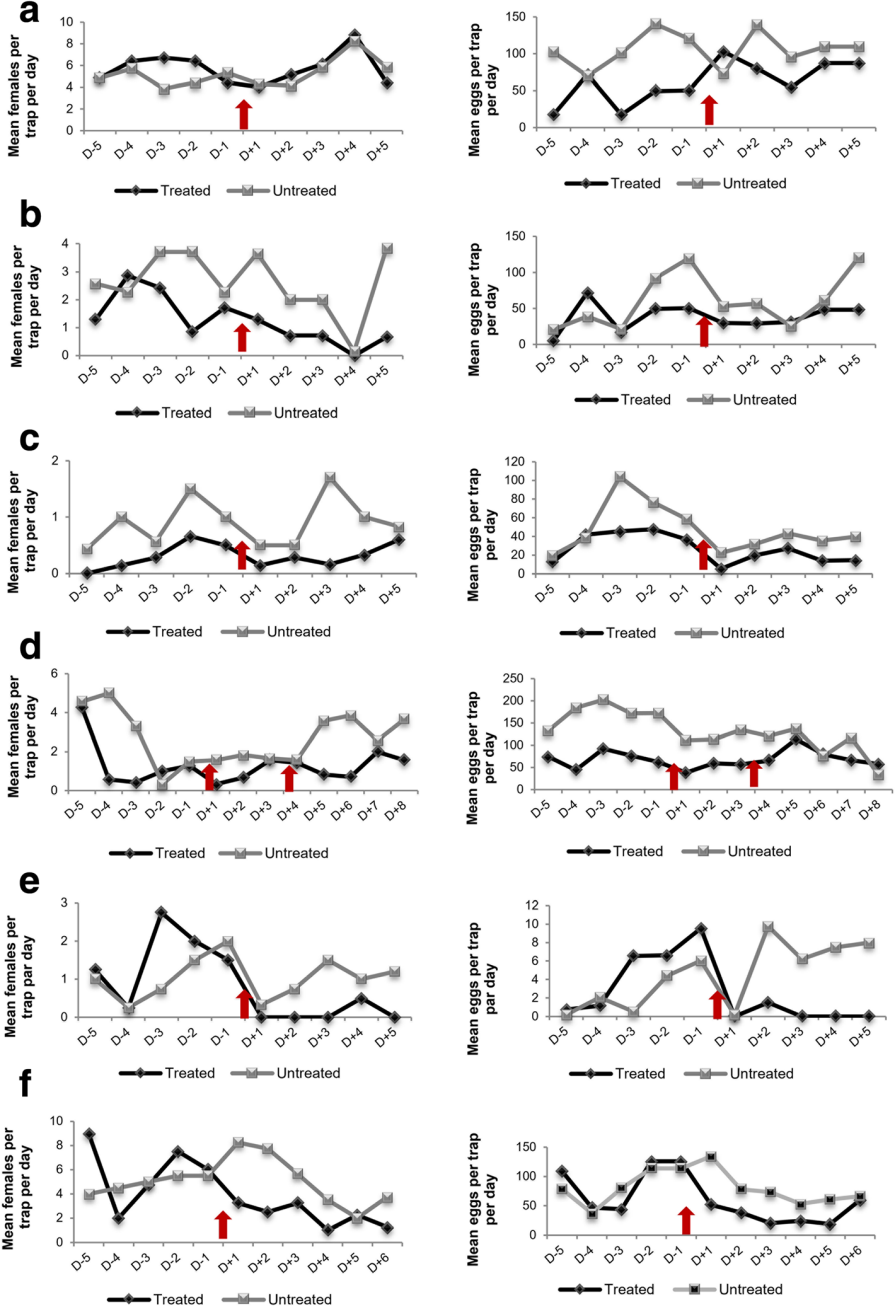
Daily number of captures of females and eggs before and after the ULV treatment (**a**–**d**) and thermal fogging treatment (**e**, **f**)

**Fig. 6:**
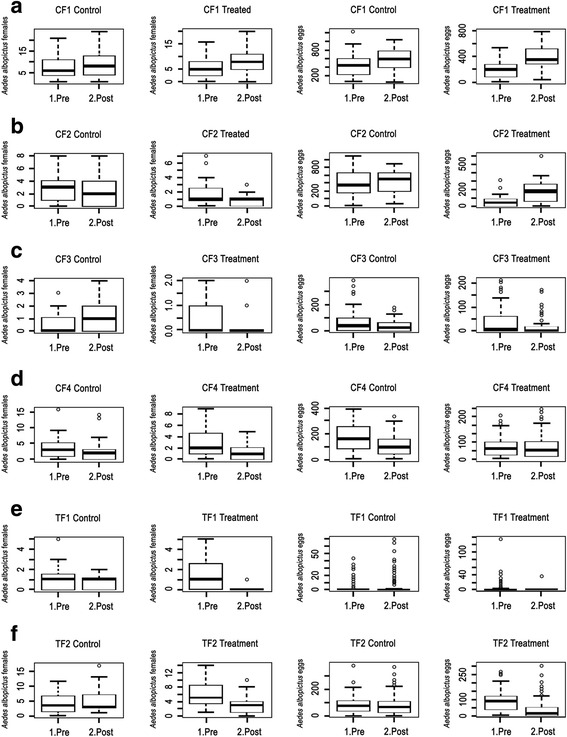
Results of the GLM binomial analysis of the influence of ULV treatment (**a**–**d**) and thermal fogging treatment (**e**, **f**) on the daily number of captures of wild females and eggs

The local strain of *Ae. albopictus* was fully susceptible to deltamethrin by both methods (Table 2); values for KdT_50_ and KdT_95_ were similar (overlapping 95% CIs) to those of the *Ae. aegypti* reference strain. We investigated the effect of the insecticidal treatment on the density and parity rates of natural populations of *Ae. albopictus*. Weather conditions appeared optimum; wind-speed was < 10 km/h and thermal conditions were stable. There was no marked impact of the fogging treatments on oviposition rate, adult capture rate or parous rate in any of the four field ULV applications (Additional file 6: Table S2; Additional file 7: Table S3; Additional file 8: Table S4; Fig. 5). Our efforts to improve the cold fogging spraying method in residential habitat was not successful despite the change to a site with a more extensive road network (Fig. 2) which assumed better coverage of the targeted zone by the swath of the insecticide cloud. Furthermore, even when two applications were made three days apart there was no impact on the wild mosquito population as shown by GLM analysis (Fig. 6) and the variable interaction “Treatment*Pre/Post” is positive and significant or not significant for all of the cases (Additional file 5: Table S1). Hence, in general Cold Fogging was not effective on diminishing the abundance of eggs and females and females’ parity rates (Additional file 6: Table S2; Additional file 7: Table S3; Additional file 8: Table S4).

**Table 2:** Deltamethrin susceptibility status of *Aedes albopictus* populations from Nice, southeastern France

Strain	Topical application				WHO test kit assay				Knockdown times			
	LD50 (ng/mg) (95% CI)	LD95 (ng/mg) (95% CI)	RR50	RR95	LD50 (%) (95% CI)	LD95 (%) (95% CI)	RR50	RR95	T50 (min) (95% CI)	KT95 (min) (95% CI)	RR50	RR95
*Aedes albopictus*	0.01147 (0.00794–0.01531)	0.05701 (0.03611–0.14515)	0.974	1.123	0.00273 (0.00228–0.00325)	0.01978 (0.01388–0.03287)	0.62	0.75	16 (15.5–16.5)	24.7 (23.2–26.6)	0.979	0.939
*Aedes aegypti (Bora)*	0.01176 (0.00849–0.01525)	0.05074 (0.03366–0.11584)	1	1	0.00131 (0.00098–0.00183)	0.00896 (0.00538–0.02008)	1	1	16.3 (15.6–17.2)	26.3 (23.8–30.3)	1	1

By contrast, in treatment by thermal fog, oviposition rate and adult capture were reduced by about 95% (Fig.5) in test 1 and 61% in Test 2; “Treatment*Pre/Post” was negative and highly significant (*P* < 10^-5^; Additional file 6: Table S2; Additional file 7: Table S3). Therefore, the thermal fogging was effective to reduce the egg and the female abundance.

Intriguingly, even if the number of eggs and adults dropped drastically there was no effect of the spraying on the female’s parity rates as shown by the GLM analysis (Additional file 4: Figure S4; Additional file 8: Table S4). The statistical power of the analysis is illustrated by the explained variance in Additional file 9: Table S5.

Temperature and air velocity measurement inside and outside the vegetation in five points chosen randomly in the sprayed zone showed that atmospheric conditions were completely different from inside than outside bushes. Results emphasize the fact that the air velocity inside the vegetation is approximately 10-fold lower that in outside (outside vegetation: 1.13–2.01 m/s; inside vegetation: 0.15–0.5 m/s).

## Discussion

In contrast to previous evaluations, nearly all of which have relied on the mortality of caged mosquitoes, we assessed the impact of treatments by monitoring the wild mosquito population on a daily (24-h) basis. As reported in other temperate regions [21–23], the local strain of *Ae. albopictus* was fully susceptible to deltamethrin. The lack of impact that we observed was in line with results obtained by a similar approach (ovitraps and back-pack aspirators) against *Ae. aegypti* in Puerto Rico, Jamaica and Venezuela [12] and in Panama [24].

Our results were disappointing because we had supposed that *Ae. albopictus* would be more vulnerable than *Ae. aegypti* because of its markedly exophilic behaviour [25]. We suggest that the lack of efficacy was simply due to lack of interaction between aerosol particles and the mosquitoes: the mosquito favors resting sites, particularly vegetation, that are devoid of air movement but the insecticide particles depend on the nuances of air movement to deliver them to the mosquito. Indeed in nearly every country, *Ae. albopictus* is common in suburban and rural situations were important vegetation is available [25]. In densely crowded urban areas which lack vegetation and outdoor breeding sites, *Ae. albopictus* may be rare or absent [26]. Using a hot-wire anemometer, we observed at least a ten-fold reduction in air movement in the interior of bushes that were abundant in the study area and it was clear that there are many sites (e.g. the leeward side of trunks, branches, leaves, crevices and other hollow structures) where mosquitoes can shelter with minimal exposure to air currents.

Apart from the lack of delivery to resting sites, there are clearly other limitations to the efficacy of ULV, particularly in urban areas, where walls, buildings and other structures obstruct the drift of particles [27]. In this context, the term “space spray” is appropriate because much of the aerosol drifts through open spaces, around or over obstacles. Moreover, mortality in a cage has little relation to mortality of resting or even free-flying mosquitoes, particularly at sites in vegetation where mosquitoes are likely to rest; in studies of the control of *Ae. aegypti* in Venezuela, mortality was more than 90% in caged mosquitoes set in the open but close to zero at typical indoor resting sites [12]. Similarly, Mount et al. [28] reported 90% mortality of caged mosquitoes in an open field but 34–67% in vegetation and Andis et al. [29] observed 95.5% mortality in caged *Ae. aegypti* suspended in the open vs. 49% in more sheltered locations. Moreover, Bengoa et al. [30] evaluated the efficacy of the ULV truck-mounted vehicle and obtained nearly 100% mortality in caged mosquitoes of *Ae. albopictus* in an open area but stated that this impact would be lower in wild uncaged mosquitoes resting within vegetation.

Logistically, ULV should be the control method of choice: a single vehicle with a driver and an operator can cover 50–80 ha in about three hours, dependent on the layout of roads and ease of access [26]. However, there is no documented evidence that ULV treatments have ever had a discernible impact on transmission of dengue or chikungunya anywhere in the world; this is not surprising, given our results.

By contrast, in our small test of thermal fog, the aerosol was applied at close quarters to the presumed resting sites, directed by the operator and boosted by the physical thrust from the exhaust energy of the machine. Therefore, hand-held thermal fogging was highly effective, eliminating between 61 and 95% of females and eggs after a single treatment directly applied in the vegetation surrounding the treated houses. These results are broadly in accordance with those of Britch et al. [31] who evaluated the efficiency of truck mounted ULV and thermal fogger and found that there is 100-fold greater chance that a droplet will come in contact with a mosquito in the sentinel cage in a thermal fog application versus a ULV application.

## Conclusions

We conclude that in the event of outbreaks of disease, truck-mounted ULV is unlikely to have significant impact on transmission but that, despite being highly labor-intensive, thermal or ULV aerosols dispensed from portable sprayers are the method of choice. Clearly this is not practicable on any large scale but may be useful in the event of potential “hot-spots” of local transmission.

## Additional files

Additional file 1: Figure S1. Route of the fogging at les Ambassades residence. (TIF 2329 kb)
Additional file 2: Figure S2. Route of the fogging at La Soubrane residence. (TIF 3141 kb)
Additional file 3: Figure S3. Route of the fogging at zone A, Les Terrasses de Saint Julien. (TIF 400 kb)
Additional file 4: Figure S4. Results of the GLMM binomial analysis of the influence of the treatments on parity rates of the natural population of *Aedes albopictus* in Nice. (TIF 5172 kb)
Additional file 5: Table S1. Biological interpretation of the variables and results of the GLMM analysis. We used the model with interactions to explain the effect of the treatment. (DOCX 11 kb)
Additional file 6: Table S2. Results of the GLMM with negative binomial distribution analysis of the influence of the treatment on egg abundance. The dependent variable is the abundance of eggs and the independent variable is pre-post treatment. (DOCX 15 kb)
Additional file 7: Table S3. Results of the GLMM with negative binomial distribution analysis of the influence of the treatment on female abundance. The dependent variable is the abundance of females and the independent variable pre-post treatment. (DOCX 15 kb)
Additional file 8: Table S4. Results of the GLMM with negative binomial distribution analysis of the influence of the treatment on parity rate. The dependent variable is parity rate and the independent variable pre-post treatment. (DOCX 15 kb)
Additional file 9: Table S5. Explained variance (deviance) for the results of the GLM analysis on the influence of treatment on the abundance of *Ae. albopictus* females and eggs. (DOCX 11 kb)

## References

[1] Bhatt S, Gething PW, Brady OJ, Messina JP, Farlow AW, Moyes CL (2013). The global distribution and burden of dengue. Nature.

[2] Musso D, Cao-Lormeau VM, Gubler DJ (2015). Zika virus: following the path of dengue and chikungunya?. Lancet.

[3] Adhami J, Reiter P (1998). Introduction and establishment of *Aedes* (*Stegomyia*) *albopictus* skuse (Diptera: Culicidae) in Albania. J Am Mosq Control Assoc.

[4] Rezza G, Nicoletti L, Angelini R, Romi R, Finarelli AC, Panning M (2007). Infection with chikungunya virus in Italy: an outbreak in a temperate region. Lancet.

[5] Septfons A, Noël H, Leparc-Goffart I, Giron S, Delisle E, Chappert JL (2015). Monitoring of chikungunya and dengue in metropolitan France. Bull Epidemiol Hebd.

[6] Delisle E, Rousseau C, Broche B, Leparc-Goffart I, L’Ambert G, Cochet A (2015). Chikungunya outbreak in Montpellier, France, September to October 2014. Eurosurveillance.

[7] Roiz D, Boussès P, Simard F, Paupy C, Fontenille D (2015). Autochthonous Chikungunya transmission and extreme climate events in southern France. PLoS Negl Trop Dis.

[8] Schaffner F, Fontenille D, Mathis A (2014). Autochthonous dengue emphasises the threat of arbovirosis in Europe. Lancet Infect Dis.

[9] Diallo D, Sall AA, Diagne CT (2014). Zika virus emergence in mosquitoes in southeastern Senegal, 2011. PLoS One.

[10] Wong PS, Li MZ, Chong CS, Ng LC (2013). Tan CH *Aedes (Stegomyia) albopictus* (Skuse): a potential vector of Zika virus in Singapore. PLoS Negl Trop Dis.

[11] Bonds JAS (2012). Ultra-low-volume space sprays in mosquito control: a critical review. Med Vet Entomol.

[12] Reiter P, Gubler DJ, Ooi EE, Vasudevan S, Farrar J (2014). Surveillance and Control of Dengue vectors. Chapter 25. Dengue and Dengue Hemorrhagic Fever.

[13] Newton EA, Reiter PA (1992). Model of the transmission of dengue fever with an evaluation of the impact of ultra-low volume (ULV) insecticide applications on dengue epidemics. Am J Trop Med Hyg.

[14] Reiter P, Amador MA, Colon N (1991). Enhancement of the CDC ovitrap with hay infusion for daily monitoring of *Ae. aegypti* populations. J Am Mosq Control Assoc.

[15] World Health Organization (2009). Guidelines for efficacytesting of insecticidesfor indoor and outdoor ground-applied space spray applications.

[16] World Health Organization (2006). Review of Dimilin^®^ GR and DT, Vectobac^®^ DT, Aqua K-Othrine^®^, Aqua Reslin Super^®^. Report of the ninth WHOPES working group meeting, 5-9 December 2005.

[17] World Health Organization (2003). Space Spray Application of Insecticides for Vector and Public Health Pest Control: a Practioner’s Guide.

[18] R Core team (2013). R: A language and environment for statistical computing.

[19] Zuur AF, Leno EN, Elphick CS, Zuur AF, Leno EN, Elphick CS (2010). A protocol for data exploration to avoid common statistical problems. Meth Ecol Evol.

[20] Bolker BM, Brooks ME, Clark CJ, Geange SW, Poulsen JR, Stevens MH, White JS (2009). Generalized linear mixed models: a practical guide for ecology and evolution. Trends Ecol Evol.

[21] Romi R, Toma L, Severini F, Di Luca M (2003). Susceptibility of Italian populations of *Aedes albopictus* to temephos and to other insecticides. J Am Mosq Control Assoc.

[22] Marcombe S, Farajollahi A, Healy SP, Clark GG, Fonseca DM (2014). Insecticide resistance status of United States populations of *Aedes albopictus* and mechanisms involved. PLoS One.

[23] Vontas J, Kioulos E, Pavlidi N, Morou E, della Torre A, Ranson H (2012). Insecticide resistance in the major dengue vectors *Aedes albopictus* and *Aedes aegypti*. Pestic Biochem Physiol.

[24] Perich MJ, Davila G, Turner A, Garcia A, Nelson M (2000). Behavior of resting *Aedes aegypti* (Culicidae: Diptera) and its relation to ultra-low volume adulticide efficacy in Panama City, Panama. J Med Entomol.

[25] Hawley WA (1988). The biology of *Aedes albopictus*. J Am Mosq Control Assoc Suppl.

[26] Rudnick A, Hammon WMD (1960). Newly recognized *Aedes aegypti* problems in Manila and Bangkok. Mosq News.

[27] Reiter P, Nathan M. Guidelines for Assessing the Efficacy of Insecticidal Space Sprays for Control of the Dengue Vector *Aedes aegypti*. Geneva: World Health Organization; 2001.

[28] Mount GA (1998). A critical review of ultralow-volume aerosols of insecticide applied with vehicle-mounted generators for adult mosquito control. J Am Mosq Control Assoc.

[29] Andis MD, Sackett SR, Carroll MK, Bordes ES (1987). Strategies for the emergency control of arboviral epidemics in New Orleans. J Am Mosq Control Assoc.

[30] Bengoa M, Eritja R, Lucientes J (2014). Ground ultra-low volume adulticiding field trials using pyrethroids against *Aedes albopictus* in the Baix Llobregat region, Spain. J Am Mosq Control Assoc.

[31] Britch SC, Linthicum KJ, Wynn WW, Walker TW, Farooq M, Smith VL (2010). Evaluation of ULV and thermal fog mosquito control applications in temperate and desert environments. J Am Mosq Control Assoc.

[32] Wilder-Smith A, Renhorn KE, Tissera H, Abu Bakar S, Alphey L, Kittayapong P, et al. DengueTools: innovative tools and strategies for the surveillance and control of dengue. Glob Health Action. 2012;22(5):17273. 10.3402/gha.v5i0.17273 PMC3312611 22451836

