# Supplementary figures and images for: Efficacy of ULV and thermal aerosols of deltamethrin for control of Aedes albopictus in nice, France
Source: Parasit Vectors. 2016 Nov 23;9:597. doi: 10.1186/s13071-016-1881-y (PMC5120493; doi:10.1186/s13071-016-1881-y)

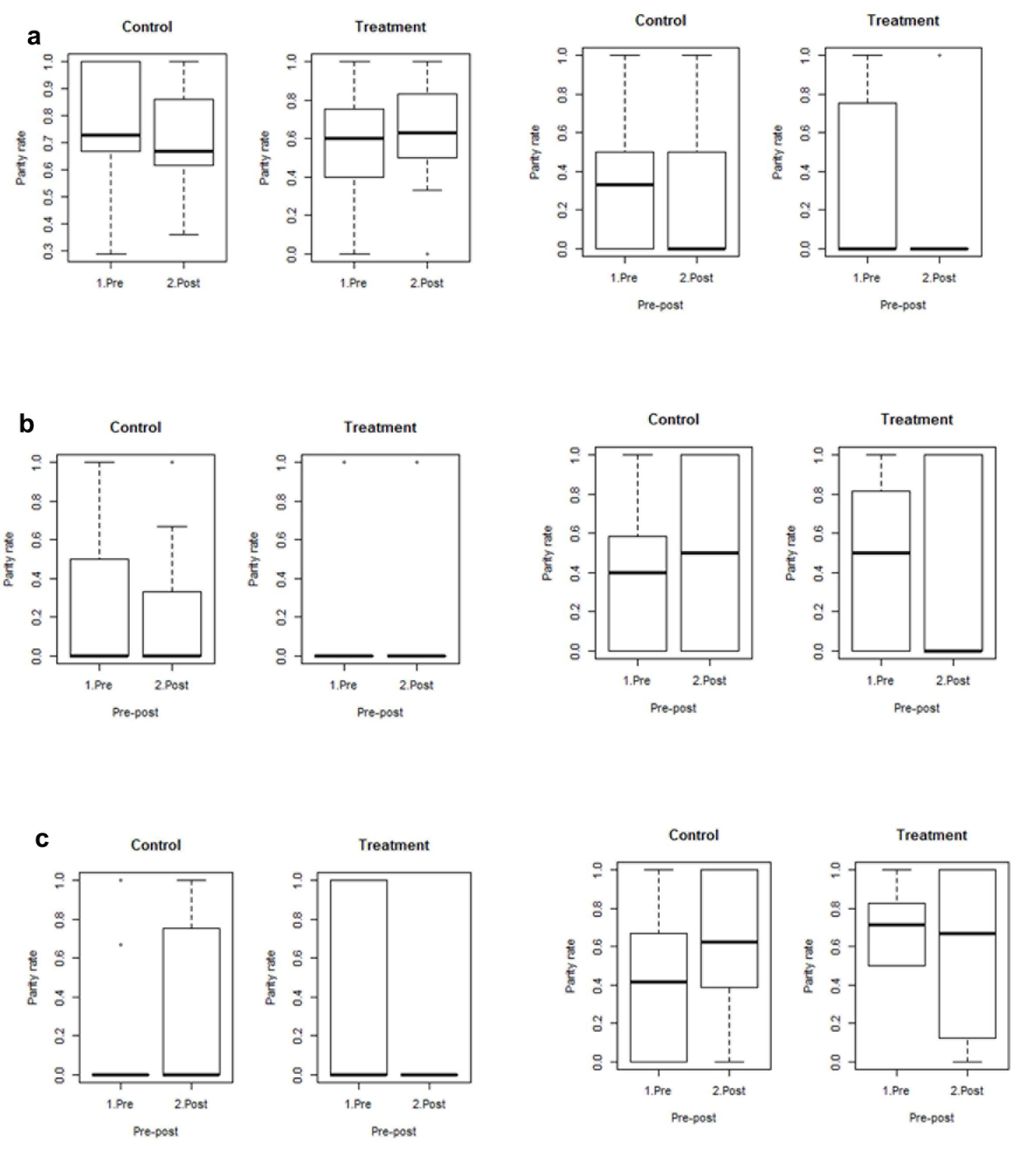

Supplement: Additional file 4: Figure S4. — Results of the GLMM binomial analysis of the influence of the treatments on parity rates of the natural population of Aedes albopictus in Nice. (TIF 5172 kb) [file 13071_2016_1881_MOESM4_ESM.tif]
