# Supplementary material for: Efficacy of ULV and thermal aerosols of deltamethrin for control of Aedes albopictus in nice, France
Source: Parasit Vectors. 2016 Nov 23;9:597. doi: 10.1186/s13071-016-1881-y (PMC5120493; doi:10.1186/s13071-016-1881-y)
Supplement: Additional file 5: Table S1. — Biological interpretation of the variables and results of the GLMM analysis. We used the model with interactions to explain the effect of the treatment. (DOCX 11 kb) [file 13071_2016_1881_MOESM5_ESM.docx]

**S5 Table: Biological interpretation of the variables and results of the GLMM Analysis.** We used the model with interactions to explain the effect of the treatment**.**

| “Treatment*Pre/Post” | | “Treatment” | | “Pre/Post” | |
| --- | --- | --- | --- | --- | --- |
| “Treatment*Pre/Post” > 0 and p **<**0.05: there is more mosquitoes after the treatment than before in the treated area, and therefore, the treatment is not effective. | “Treatment*Pre/Post” **<** 0 and p **<**0.05: there is less mosquitoes after the treatment than before in the treated area, and therefore, the treatment is effective. | “Treatment” > 0 and p **<**0.05: the treated area has more mosquitoes than the control area. | “Treatment” **<** 0 and p **<**0.05: the treated area has less abundance than the control area. | “Pre/Post” > 0 and p **<**0.05: the “Post” area has more abundance that the “Pre” area. | “Pre/Post” **<** 0 and p **<**0.05: the “Pre” area has more abundance that the “Post” area. |
