# Supplementary material for: Efficacy of ULV and thermal aerosols of deltamethrin for control of Aedes albopictus in nice, France
Source: Parasit Vectors. 2016 Nov 23;9:597. doi: 10.1186/s13071-016-1881-y (PMC5120493; doi:10.1186/s13071-016-1881-y)
Supplement: Additional file 6: Table S2. — Results of the GLMM with negative binomial distribution analysis of the influence of the treatment on egg abundance. The dependent variable is the abundance of eggs and the independent variable is pre-post treatment. (DOCX 15 kb) [file 13071_2016_1881_MOESM6_ESM.docx]

**Additional file 6: Table S2.** Results of the GLMM with negative binomial distribution analysis of the influence of the treatment on egg’s abundance. The dependent variable is the abundance of eggs and the independent variable is pre-post treatment.

| Spraying method | Test | Variables | Estimate | Standard error | Z value | p |
| --- | --- | --- | --- | --- | --- | --- |
| Cold fogging | CF1 | Intercept | 6.161 | 0.133 | 46.41 | < 2e-16 |
|  |  | Treatment | -0.865 | 0.178 | -4.86 | 1.1e-06 |
|  |  | Pre/post | 0.190 | 0.177 | 1.07 | 0.282 |
|  |  | Treatment*Pre/Post | 0.471 | 0.249 | 1.89 | 0.058 |
|  | CF2 | Intercept | 5.6776 | 0.2087 | 27.21 | < 2e-16 |
|  |  | Treatment | -1.4922 | 0.2959 | -5.04 | 4.6e-07 |
|  |  | Pre/post | 0.0831 | 0.2951 | 0.28 | 0.778 |
|  |  | Treatment*Pre/Post | 0.9600 | 0.4180 | 2.30 | 0.022 |
|  | CF3 | Intercept | 4.089 | 0.207 | 19.76 | < 2e-16 |
|  |  | Treatment | -0.477 | 0.293 | -1.63 | 0.10 |
|  |  | Pre/post | -0.555 | 0.295 | -1.88 | 0.06 |
|  |  | Treatment*Pre/Post | -0.270 | 0.415 | -0.65 | 0.52 |
|  | CF4 | Intercept | 5.151 | 0.095 | 54.24 | < 2e-16 |
|  |  | Treatment | -0.912 | 0.135 | -6.75 | 1.5e-11 |
|  |  | Pre/post | -0.493 | 0.121 | -4.08 | 4.6e-05 |
|  |  | Treatment*Pre/Post | 0.458 | 0.172 | 2.66 | 0.0077 |
| Thermal Fogging | TF1 | Intercept | 0.468 | 0.566 | 0.83 | 0.41 |
|  |  | Treatment | 0.858 | 0.580 | 1.48 | 0.14 |
|  |  | Pre/post | 0.511 | 0.549 | 0.93 | 0.35 |
|  |  | Treatment*Pre/Post | -5.768 | 1.051 | -5.49 | 4.1e-08 |
|  | TF2 | Intercept | 4.4093 | 0.1223 | 36.05 | < 2e-16 |
|  |  | Treatment | 0.0805 | 0.1552 | 0.52 | 0.60 |
|  |  | Pre/post | -0.0855 | 0.1478 | -0.58 | 0.56 |
|  |  | Treatment*Pre/Post | -0.9015 | 0.2100 | -4.29 | 1.8e-05 |
