# Supplementary material for: Efficacy of ULV and thermal aerosols of deltamethrin for control of Aedes albopictus in nice, France
Source: Parasit Vectors. 2016 Nov 23;9:597. doi: 10.1186/s13071-016-1881-y (PMC5120493; doi:10.1186/s13071-016-1881-y)
Supplement: Additional file 7: Table S3. — Results of the GLMM with negative binomial distribution analysis of the influence of the treatment on female abundance. The dependent variable is the abundance of females and the independent variable pre-post treatment. (DOCX 15 kb) [file 13071_2016_1881_MOESM7_ESM.docx]

**Additional file 7: Table S3.** Results of the GLMM with negative binomial distribution analysis of the influence of the treatment on female’s abundance. The dependent variable is the abundance of females and the independent variable pre-post treatment.

| Spraying method | Test | Variables | Estimate | Standard error | Z value | p |
| --- | --- | --- | --- | --- | --- | --- |
| Cold fogging | CF1 | Intercept | 2.068 | 0.115 | 18.02 | <2e-16 |
|  |  | Treatment | -0.367 | 0.178 | -2.07 | 0.039 |
|  |  | Pre/post | 0.110 | 0.159 | 0.69 | 0.489 |
|  |  | Treatment*Pre/Post | 0.306 | 0.236 | 1.30 | 0.195 |
|  | CF2 | Intercept | 1.038 | 0.153 | 6.79 | 1.1e-11 |
|  |  | Treatment | -0.440 | 0.203 | -2.17 | 0.030 |
|  |  | Pre/post | -0.230 | 0.195 | -1.18 | 0.240 |
|  |  | Treatment*Pre/Post | -0.764 | 0.336 | -2.27 | 0.023 |
|  | CF3 | Intercept | 0.0724 | 0.3035 | 0.24 | 0.8113 |
|  |  | Treatment | -1.0666 | 0.3914 | -2.73 | 0.0064 |
|  |  | Pre/post | 0.0466 | 0.2949 | 0.16 | 0.8745 |
|  |  | Treatment*Pre/Post | -0.0464 | 0.5668 | -0.08 | 0.9347 |
|  | CF4 | Intercept | 1.286 | 0.196 | 6.57 | 4.9e-11 |
|  |  | Treatment | -0.266 | 0.228 | -1.17 | 0.243 |
|  |  | Pre/post | -0.336 | 0.213 | -1.58 | 0.114 |
|  |  | Treatment*Pre/Post | -0.519 | 0.312 | -1.66 | 0.096 |
| Thermal Fogging | TF1 | Intercept | 0.178 | 0.375 | 0.47 | 0.6349 |
|  |  | Treatment | 0.360 | 0.321 | 1.12 | 0.2621 |
|  |  | Pre/post | -0.221 | 0.379 | -0.58 | 0.0028 |
|  |  | Treatment*Pre/Post | -2.484 | 0.830 | -2.99 | 0.5602 |
|  | TF2 | Intercept | 1.399 | 0.211 | 6.62 | 3.7e-11 |
|  |  | Treatment | 0.369 | 0.226 | 1.63 | 0.1022 |
|  |  | Pre/post | 0.191 | 0.219 | 0.87 | 0.3831 |
|  |  | Treatment*Pre/Post | -0.917 | 0.317 | -2.89 | 0.0038 |
