# Supplementary material for: Efficacy of ULV and thermal aerosols of deltamethrin for control of Aedes albopictus in nice, France
Source: Parasit Vectors. 2016 Nov 23;9:597. doi: 10.1186/s13071-016-1881-y (PMC5120493; doi:10.1186/s13071-016-1881-y)
Supplement: Additional file 8: Table S4. — Results of the GLMM with negative binomial distribution analysis of the influence of the treatment on parity rate. The dependent variable is parity rate and the independent variable pre-post treatment. (DOCX 15 kb) [file 13071_2016_1881_MOESM8_ESM.docx]

**Additional file 8: Table S4.** Results of the GLMM with negative binomial distribution analysis of the influence of the treatment on parity rate. The dependent variable is parity rate and the independent variable pre-post treatment.

| Spraying method | Test | Variables | Estimate | Standard error | Z value | p |
| --- | --- | --- | --- | --- | --- | --- |
| Cold fogging | CF1 | Intercept | -0.2732 | 0.1998 | -1.37 | 0.17 |
|  |  | Treatment | -0.3343 | 0.3120 | -1.07 | 0.28 |
|  |  | Pre/post | -0.0576 | 0.2824 | -0.20 | 0.84 |
|  |  | Treatment*Pre/Post | 0.2398 | 0.4269 | 0.56 | 0.57 |
|  | CF2 | Intercept | -1.10876 | 0.29438 | -3.77 | 0.00017 |
|  |  | Treatment | -0.00431 | 0.41677 | -0.01 | 0.99175 |
|  |  | Pre/post | -0.28237 | 0.45271 | -0.62 | 0.53281 |
|  |  | Treatment*Pre/Post | -2.13091 | 1.13670 | -1.87 | 0.06084 |
|  | CF3 | Intercept | -1.4879 | 0.3814 | -3.90 | 9.6e-05 |
|  |  | Treatment | -0.2215 | 0.5485 | -0.40 | 0.69 |
|  |  | Pre/post | -0.0658 | 0.5440 | -0.12 | 0.90 |
|  |  | Treatment*Pre/Post | -0.5671 | 0.8934 | -0.63 | 0.53 |
|  | CF4 | Intercept | -0.983 | 0.294 | -3.35 | 0.00082 |
|  |  | Treatment | 0.186 | 0.387 | 0.48 | 0.63127 |
|  |  | Pre/post | 0.305 | 0.352 | 0.87 | 0.38700 |
|  |  | Treatment*Pre/Post | -0.503 | 0.489 | -1.03 | 0.30329 |
| Thermal Fogging | TF1 | Intercept | -1.963 | 0.612 | -3.21 | 0.0013 |
|  |  | Treatment | 1.047 | 0.707 | 1.48 | 0.1387 |
|  |  | Pre/post | 0.724 | 0.746 | 0.97 | 0.3322 |
|  |  | Treatment*Pre/Post | -18.475 | 2594.700 | -0.01 | 0.9943 |
|  | TF2 | Intercept | -0.870 | 0.346 | -2.52 | 0.012 |
|  |  | Treatment | 0.532 | 0.440 | 1.21 | 0.226 |
|  |  | Pre/post | 0.380 | 0.436 | 0.87 | 0.383 |
|  |  | Treatment*Pre/Post | -0.589 | 0.583 | -1.01 | 0.312 |
