# Supplementary material for: Efficacy of ULV and thermal aerosols of deltamethrin for control of Aedes albopictus in nice, France
Source: Parasit Vectors. 2016 Nov 23;9:597. doi: 10.1186/s13071-016-1881-y (PMC5120493; doi:10.1186/s13071-016-1881-y)
Supplement: Additional file 9: Table S5. — Explained variance (deviance) for the results of the GLM analysis on the influence of treatment on the abundance of Ae. albopictus females and eggs. (DOCX 11 kb) [file 13071_2016_1881_MOESM9_ESM.docx]

**Additional file 9: Table S5.** Explained variance (deviance) for the results of the GLM analysis on the influence of treatment on *Ae. albopictus* female and egg abundance.

| Test | Explained variance for females | Explained variance for eggs |
| --- | --- | --- |
| CF1^a^ | 6.13% | 22.7% |
| CF2 | 19.35% | 19.5% |
| CF3 | 12.2% | 3.7% |
| CF4 | 11.8% | 9.42% |
| TF1^b^ | 25.9% | 11.3% |
| TF2 | 10.3% | 6.9% |

^a^ CF: Cold Fogging; ^b^ TF: Thermal Fogging
